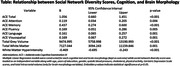# The Effect of Social Isolation on Cognitive Performance and Brain Morphology among the Urban Elderly Indian Cohort: A Cross‐Sectional Analysis

**DOI:** 10.1002/alz.090250

**Published:** 2025-01-09

**Authors:** Abhishek Mensegere Lingegodwa, Sadhana Singh, Albert Stezin, Ajith Partha, Divya N M, Amitha C M, Deva Kumar HS, Rajitha Narayanasamy, Meghana R, Meenakshi Menon, Vindhya vishwanath, Sunitha HS, Goutham Velavarajan, Prathima Arvind, Deepashri Agrawal, Palash K Malo, Banashree Mondal, Shafeeq K Shahul Hameed, Jonas S. Sundarakumar, Thomas Gregor Issac

**Affiliations:** ^1^ Centre for Brain Research, Indian Institute of Science, Bangalore, Karnataka India; ^2^ Centre for Brain Research, Bangalore, Karnataka India

## Abstract

**Background:**

Social isolation among the elderly is increasing and its detrimental effect on cognition is not fully understood. We aimed to study the effects of social isolation on cognitive performance and brain morphology among the urban elderly.

**Method:**

In this cross‐sectional analysis of 1484 participants from the Tata Longitudinal Study of Aging (TLSA) based in urban Bangalore, we administered the social network index questionnaire to identify participants experiencing social isolation. Cognitive performance was assessed using the Addenbrooke Cognitive Examination III (ACE III) battery and brain cortical volumes were obtained after subcortical volumetry was performed on T1 images (Siemens 3T Magnetom Prisma Fit) using the Freesurfer software (v 7.2.0). Multiple linear regression analysis was performed with social network diversity score (low scores indicating less involvement in social roles) as the independent variable and, ACE III total, subdomains, and brain volumes as dependent variables controlling for age, gender, education, blood pressure, cholesterol, physical activity, and depression were used as covariates.

**Result:**

In this study 727 (49%) were males, the mean age (SD) was 62.6 (9.6), the majority of them belonged to upper and middle socio‐economic strata (96.3%) and the mean (SD) education was 14 (4.2) years. People who have poor social network diversity scores performed poorly in ACE total (β=1.056, p<0.001), attention (β=0.119, p=0.006), memory (β=0.437, p<0.001), fluency (β=0.169, p=0.005), language (β=0.161, p=0.001), and visuospatial domain (β=0.169, p=0.001) (Table 1). Those with lower social network scores had lower total grey matter volume (β=9474.99, p<0.001), and higher white matter hyperintensities (β=‐0.469, p<0.001).

**Conclusion:**

Social isolation negatively affects global cognition and its subdomains and shows significant changes in brain volumes. Addressing social isolation among the elderly using policy measures should be mandated to decrease the load of cognitive impairment in the population.